# *"GenotypeColour™"*: colour visualisation of SNPs and CNVs

**DOI:** 10.1186/1471-2105-10-49

**Published:** 2009-02-04

**Authors:** Sergio Barlati, Sergio Chiesa, Chiara Magri

**Affiliations:** 1 Division of Biology and Genetics, Department of Biomedical Sciences and Biotechnology, Brescia University, Viale Europa 11, 25123 Brescia, Italy

## Abstract

**Background:**

The volume of data available on genetic variations has increased considerably with the recent development of high-density, single-nucleotide polymorphism (SNP) arrays. Several software programs have been developed to assist researchers in the analysis of this huge amount of data, but few can rely upon a whole genome variability visualisation system that could help data interpretation.

**Results:**

We have developed *GenotypeColour™ *as a rapid user-friendly tool able to upload, visualise and compare the huge amounts of data produced by Affymetrix Human Mapping GeneChips without losing the overall view of the data.

Some features of *GenotypeColour™ *include visualising the entire genome variability in a single screenshot for one or more samples, the simultaneous display of the genotype and Copy Number state for thousands of SNPs, and the comparison of large amounts of samples by producing "consensus" images displaying regions of complete or partial identity. The software is also useful for genotype analysis of trios and to show regions of potential uniparental disomy (UPD). All information can then be exported in a tabular format for analysis with dedicated software. At present, the software can handle data from 10 K, 100 K, 250 K, 5.0 and 6.0 Affymetrix chips.

**Conclusion:**

We have created a software that offers a new way of displaying and comparing SNP and CNV genomic data. The software is available free at  and is especially useful for the analysis of multiple samples.

## Background

Data visualisation is important for many scientific disciplines, and this is particularly so in genetics, where the recent development of high-density, single-nucleotide polymorphism (SNP) arrays can provide information on thousands of SNPs and structural variants [1]. Although some results can be recovered by sophisticated analysis, visual data can provide an extraordinary amount of useful information. Several programs have been developed to assist researchers in the analysis of the huge amount of data available on genetic variations. The Affymetrix software GTYPE and the more recent Genotyping Console allow raw intensity data to be analysed and provide information on genotype and intensity for each probe, which is then simple to export for analysis with other tools.

The CNAG [2] elaborates the raw data obtained by the Affymetrix platform through a simple graphical interface, and displays information on intensity and on loss-of-heterozygosity (LOH) combined with the chromosome ideogram. Another program, dChip [3], analyses raw Affymetrix data and displays the genotype, LOH and intensity information in an array of samples (columns) × SNPs (rows) with a colour assigned to each polymorphism according to a general scheme. SNPscan [4] displays genotype and Copy Number (CN) state information simultaneously to identify chromosomal abnormalities.

Our goal was to develop a user-friendly visualisation tool that could rapidly upload, visualise and compare the huge amounts of data produced by the Affymetrix GeneChip^® ^Human Mapping chips, and integrate certain earlier software functions. The visualisation tool translates the genotype and CN information obtained by the Affymetrix GeneChip^® ^Human Mapping chips into a colour code. The software allows the user to look at whole-genome variability by visualising the genotype and normalized quantitative data of one or more individuals for millions of SNPs in different colours in a single window. The entire genome SNPs can be displayed either together or separately for each chromosome.

## Implementation

*GenotypeColour*™ analyses the data produced by all of the commercially available Affymetrix GeneChip^® ^Human Mapping arrays, including the most recent 6.0 chips.

*GenotypeColour™ *is written in Visual Basic and uses the Access database to store information. If the Microsoft Access application is not installed on a computer, the program can run after the installation of Microsoft Jet 4.0 Database Engine and Microsoft Data Access Components (MDAC) 2.8 SP1, which are free to download from the Microsoft web site [5,6]. At present, *GenotypeColour™*runs only under Windows with Framework.net 1.0 and has been tested on the Windows 2000, Windows XP and Vista operating systems. The minimal hardware requirements for analysis of the 250 K arrays (and inferior) are 250 MB of RAM, and a CPU Pentium 4 1.6 GHz, whereas at least 1 GB of RAM is recommended for the analysis of 5.0 and 6.0 chips.

Software performance was tested on a notebook computer with CPU IntelCore2, 1.66 GHz and 1 GB of RAM. Uploading 250 K probes for 20 samples took 2 minutes for the genotype data and another 2 minutes for the CN state information. Visualisation of the data, however, took only 12 seconds.

The upload and visualisation of 6.0 data took slightly longer than it did for the 250 K probes, requiring almost 3 minutes for one sample and for CN state uploading, whereas visualisation of the data took approximately 1.5 minutes. However, since the longest time required was to upload the relative information of the probes' positions, the upload of the other samples was much faster. As an example, uploading the genotype data of the other 5 samples took only 3 minutes. Once the data were uploaded, opening the files took only a few seconds.

## Results

### Software description

*GenotypeColour™ *does not elaborate the raw data of the Affymetrix Human mapping chip; instead, it imports the data analysed by the Affymetrix consoles and displays them in a different way. The algorithm needs two input files, one containing genotype information and the other the CN state information. Both files are obtained by Affymetrix GTYPE or Genotyping Console Software programs. *GenotypeColour™ *is programmed to display the genomic variation in four different ways, as described below.

#### Visualisation of genotypes and the CN state

The algorithm reads the genotype SNP calls exported by Affymetrix GTYPE or GenotypeConsole software and assigns a square (down to a single pixel) to each SNP on the computer screen. All SNPs are displayed one after the other horizontally, from the top to the bottom of the page in a left-to-right pattern, starting from the first SNP on chromosome 1 and ending with the last SNP on chromosome X. For the 6.0 data, SNPs on chromosome Y and on mtDNA are displayed after those on chromosome X.

**Figure 1:**
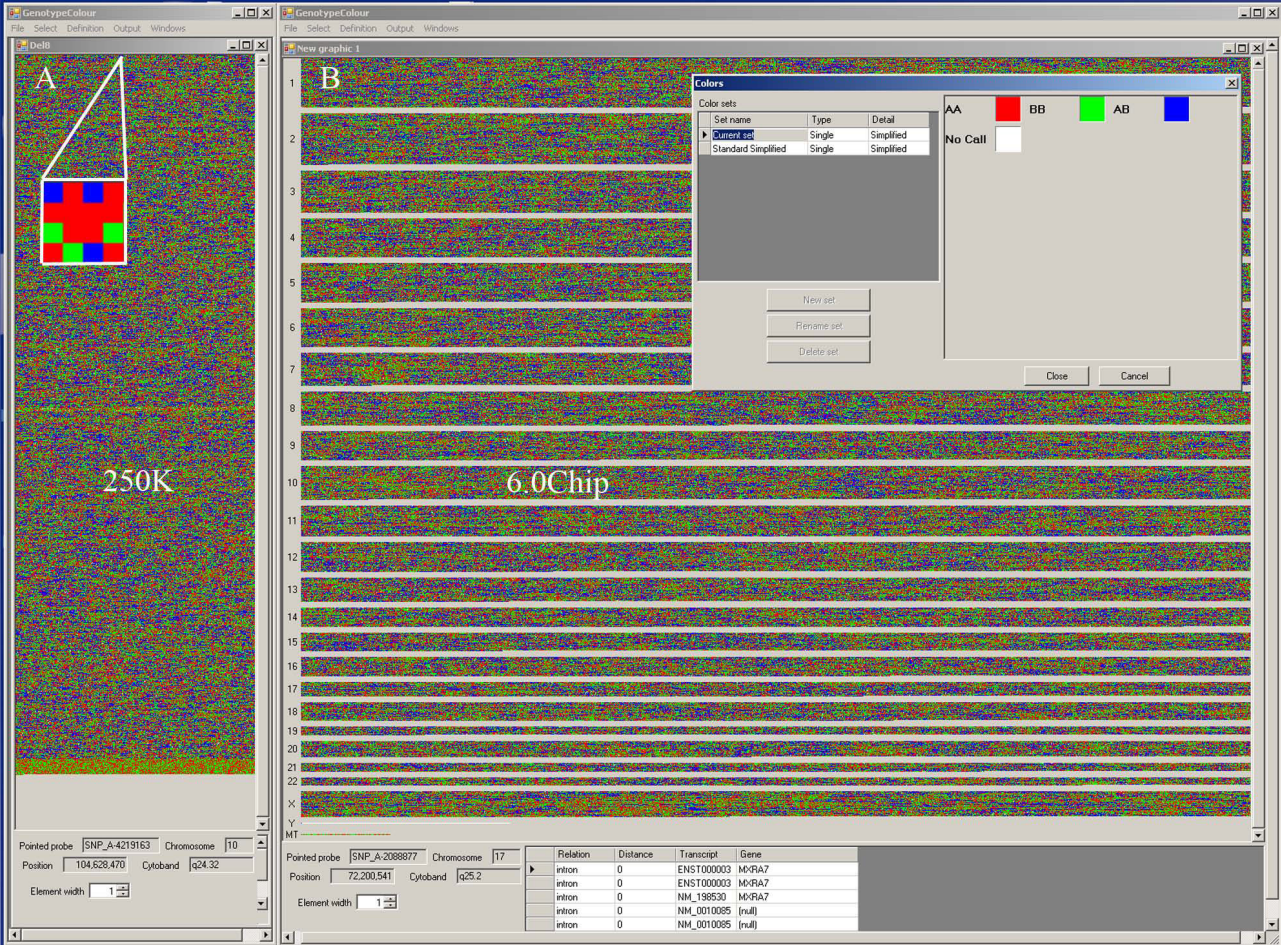
***GenotypeColour™ *whole genome visualisation**. *GenotypeColour™ *visualisation of the whole genotype variability of two samples; one analyzed with the Affymetrix Human Mapping 250 K NspI array (A) and the other with the 6.0 array (B). In the magnified visualisation of a portion of the genotype variability of sample A, we can appreciate that the blob of colours of the main figure is the result of coloured squares each representing a single SNP. Red, green, blue and white have been assigned to the AA, BB, AB and No Call genotypes, respectively. In sample B, the "separate chromosomes option" is activated and the SNPs on the different chromosomes are separated by clear lines. The name of the chromosome is reported on the left-hand side of the window. To identify a SNP, its chromosome position, cytoband and position relative to flanking genes, it is sufficient to click on the corresponding square and all this information will be visualised at the bottom of the figure, as shown for SNP_A-2088877. Note that sample A and B correspond to male and female DNA, respectively.

The number of SNPs in each line can vary from 1 to a number depending on the software window and the computer screen. It is possible to change the way the data are visualised to generate the most informative image by changing the window size. Each square is displayed with a different customised colour according to the genotype of the SNP shown (AA, BB, AB, No Call). In this way, the whole genotype variability of one subject can be visualised on the computer screen at the same time (Figure 1).

**Figure 2:**
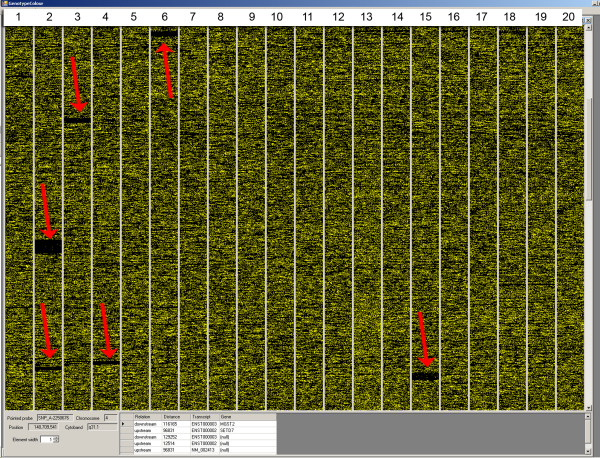
**Genotype visualisation screenshot**. A portion (3p14 – 7p15) of the genotype variation of 20 subjects is displayed. Each column, numbered 1 – 20, represents one DNA sample. In this example, to visualise LOH regions better, yellow was assigned to heterozygous genotypes and black was assigned to both homozygous genotypes. The LOH regions are highlighted by the arrows, and their number and extension are correlated with population demography.

The software allows the simultaneous display of multiple samples (Figure 2), which are shown next to one another and are visualised as coloured columns. Increasing the number of samples reduces the number of SNPs that can be visualised on the screen. The scrolling bar can be used to visualise all SNPs along the genome analysed. The genotype images can be sorted by chromosome and magnified to see the areas of interest better. By clicking on an individual SNP/square, it is possible to obtain information on its name, its genome position and its proximity to genes and coding regions. The graphical visualisation of genotypes can be transformed into a table displaying the percentages of AA, BB, AB and No Call for each sample in the whole genome or in a selected region, by selecting the "show statistics" option.

The use of customized colours to visualise data is very useful for the detection of particular regions. For example, in Figure 2, which displays a portion of the genome variability of 20 subjects, the attribution of the same colour (black) to the homozygous genotypes allows LOH regions to be visualised better. To help detect these areas, it is possible to create a list of all chromosome regions with more than *n *(where *n *is a customised number) consecutive SNPs with/without a particular genotype. This list can be exported in an Excel format.

The SNP allele frequencies of European, Asian and African populations as reported by the Affymetrix database are memorised in the software for all chips, excluding 6.0. After selecting a population for each subject, the software assigns a colour to each homozygous genotype according to whether it is the most frequent or the rarest homozygote in that population. The percentage of rare homozygotes is also displayed, and this may help the classification of samples.

**Figure 3:**
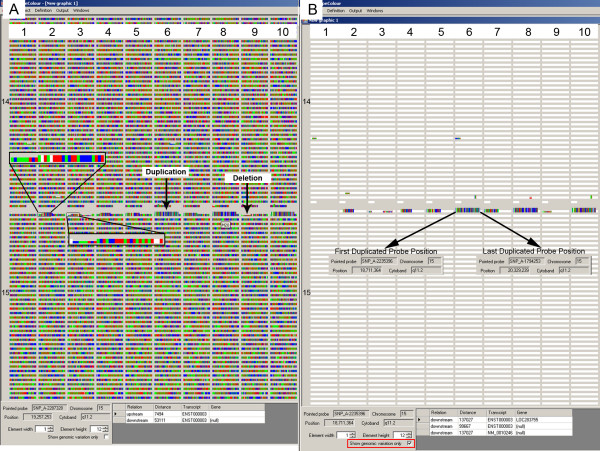
**Genotype and CN state visualisation**. The portions of chromosome 14 and 15 of 10 subjects numbered 1 – 10, are shown. A) Genotype and CN state visualisation mode. As seen better in the magnified portion, the colour of the squares depends on the SNP genotype, whereas their height is proportional to their CN states. A deletion and a duplication are shown by arrows. B) "Show genomic variation only" visualisation mode. When this option (shown by a red rectangle at the bottom of the page) is selected, all SNPs with a CN state equal to state 2 are turned into white to improve the visualisation of deletions and duplications only. To show the chromosomal position of one of the visualised duplications, the information about the position of the first and last duplicated probes is reported. This information is usually displayed at the bottom of the window by clicking on the region of interest.

After importing the CN state of each probe from the Affymetrix CNAT4 viewer or Genotyping Console, the information can be displayed immediately with the genotype information. Whereas the genotype information is given by the colour of the square, the CN state (0, 1, 2, 3 and 4 for each allele) is displayed by changing the height of the squares, thus visualising genotype and CN state simultaneously (Figure 3A). Obviously, changing only the height of the squares converts them into rectangles. In this visualisation, both the polymorphic and the non-polymorphic probes are displayed for the 6.0 chips, with the difference that the former are coloured according to genotype, whereas the latter have the same colour as the No Call genotype.

This option may be useful to test the accuracy of the data. For example, the visualisation of heterozygous SNPs in regions with a CN state equal to 1 could suggest genotype error, CN miscalculation, hybridization of probes to segmental duplicated regions or mosaicism. To visualise CN alterations (CNAs) and the smaller CN variations (CNVs) dispersed within the genome better, it is possible to select the "show genomic variation only" option, which allows the visualisation of regions with CN states other than 2, by masking all other information (Figure 3B).

**Figure 4:**
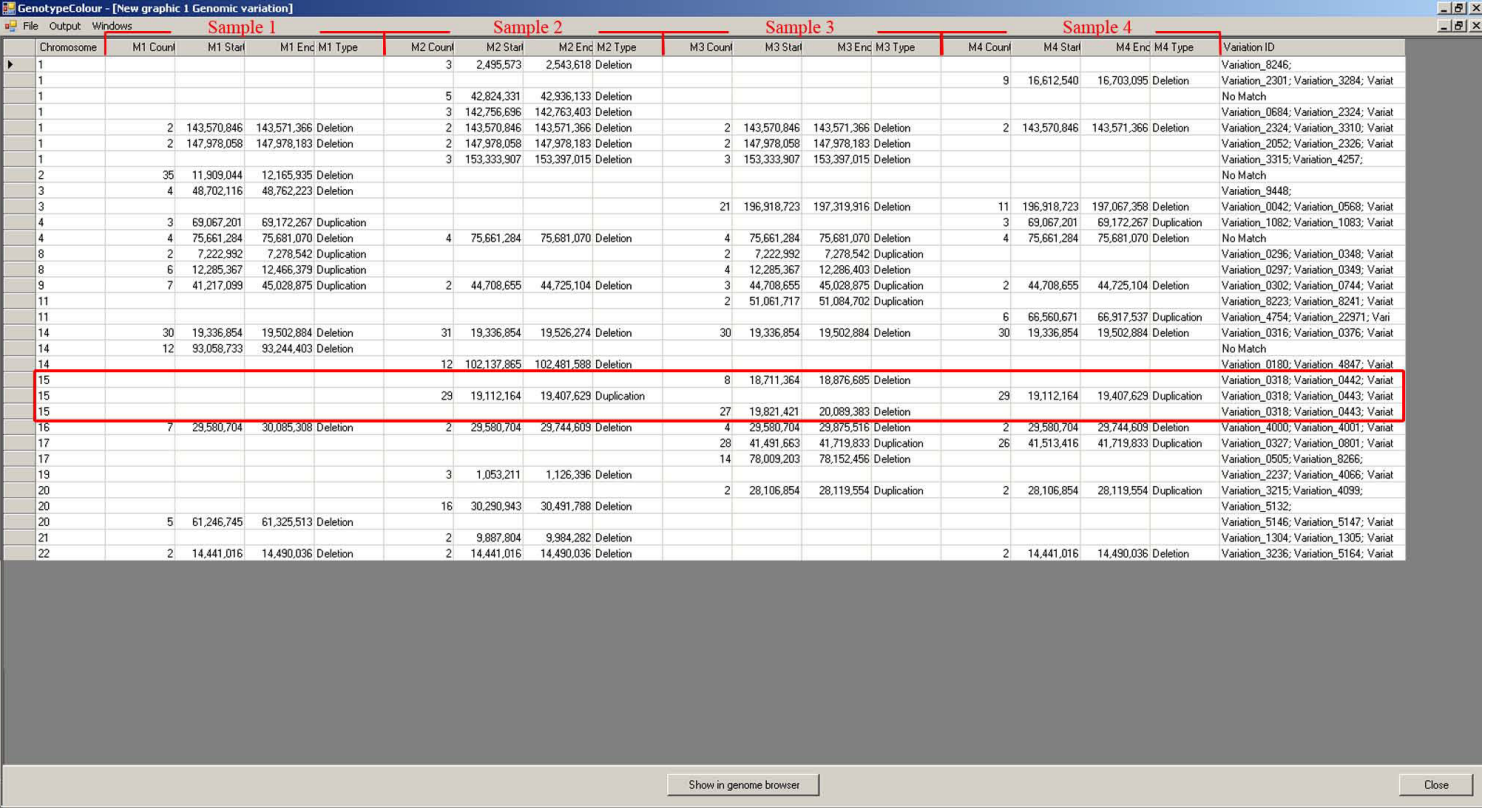
**Genomic CNV table**. List of all CNVs identified in four samples. All the compared samples are reported in the same table. Each sample is represented by four columns (from column 2 to the second-last): first column, the number of SNPs involved in the CNV; second column, the start position (in bp) of the CNV; third column, the end position of the CNV; fourth column, the type of CNV (deletion or duplication). The CNVs, identified in different samples that share at least a partially overlapping fragment, are reported in the same row. So, each row represents a different CNV and the number of rows corresponds to the number of different CNVs identified in all compared samples. The first column of the table reports the chromosome where the CNVs are located, the last column reports the name of the equivalent variations in the Database of Genomic Variants (DGV) [7], when present. The three CNVs on chromosome 15 of the first four samples of Figure 3 are highlighted by a red square.

In this visualisation, the software allows the user to create a list of chromosomal regions with a CN state other than 2 (Figure 4). The CNVs are displayed in a table of samples (columns) and CNVs (rows). This representation helps to identify CNVs shared among samples, since overlapping CNVs appear in the same row. For each CNV, its chromosome position, the number of SNPs involved, the equivalent variation in the Database of Genomic Variants [7] and an active link to the UCSC Human Genome Browser Database [8] are displayed. The CNV list can be exported into an Excel format.

#### Genotype comparison

The other important feature of *GenotypeColour™ *is its sample genotype comparison function. Presently, the software allows the genotype or the CN state of many individuals for thousands of SNPs to be depicted, and it allows comparisons to be shown.

When comparing two or more samples, for each SNP the algorithm evaluates whether the samples share the same genotype, only one allele or no allele, and assigns a colour to the corresponding square on the screen. This function allows fast and simple detection of chromosomal regions of complete or partial identity among samples, and allows identification of the origin of chromosomal alteration in parent-child comparison. A list of these regions can be exported in Excel format. Graphical visualisation is combined with information on the percentage of SNPs sharing identical genotypes, only one allele or no allele.

One implementation of genotype comparison is trio comparison. When this option is selected, the software displays SNPs with Mendelian segregation and with Mendelian inconsistencies in two different colours.

#### CN state comparison

**Figure 5:**
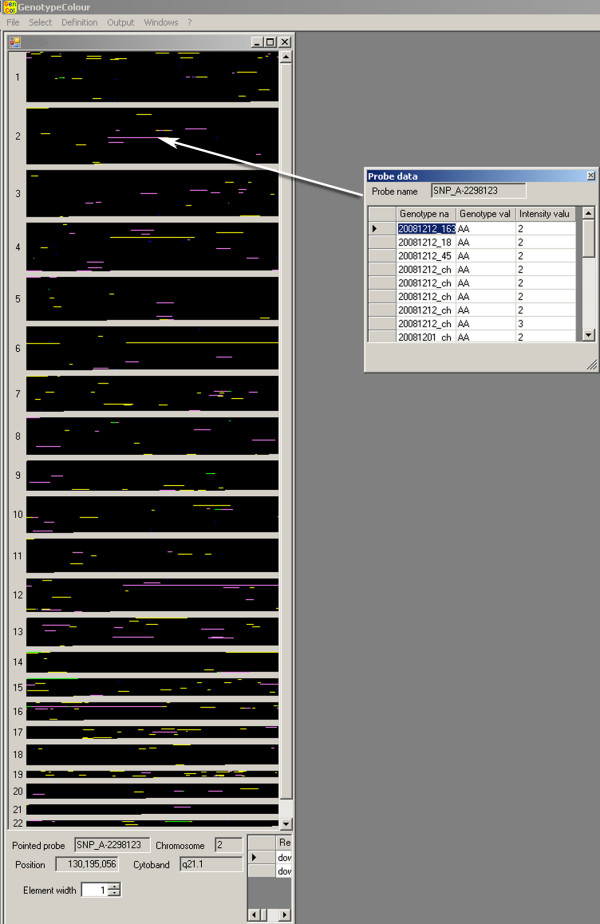
**Screenshot of CN state comparison visualisation mode**. The CN state comparison of 30 healthy subjects is reported. Only the CN state comparison of autosome chromosomes is shown. SNPs with a diploid state (CN state 2) in all samples are in black; SNPs with a CN state lower than 2 (deleted) in all the samples are in blue; SNPs with a CN state lower than 2 in only some samples are in yellow; SNPs with a CN state greater than 2 (duplicated) in all the samples are in red; SNPs with a CN state greater than 2 in only some samples are in pink; and SNPs that were simultaneously duplicated and deleted in different samples are in green. In this figure, a duplication (pink) common to some samples is highlighted by an arrow. The position of one of the SNPs belonging to the highlighted deletion is reported at the bottom of the window. In the figure, a "Probe data" window is also reported. This window can be opened by double-clicking on any SNPs/squares visualised, and it displays the intensity value and the genotype of all compared samples for that particular SNP.

As well as genotype comparison, *GenotypeColour™ *allows the user to compare the CN state of two or more samples. In this case, the algorithm compares the CN state of each SNP in all samples analysed and assigns each SNP a colour depending on the outcome of the comparison. For each SNP, there are six possible outcomes: a diploid state in all samples; deleted or duplicated in all samples; deleted in some samples; duplicated in some samples; and deleted and duplicated in some samples (Figure 5).

### Applications

**Figure 6:**
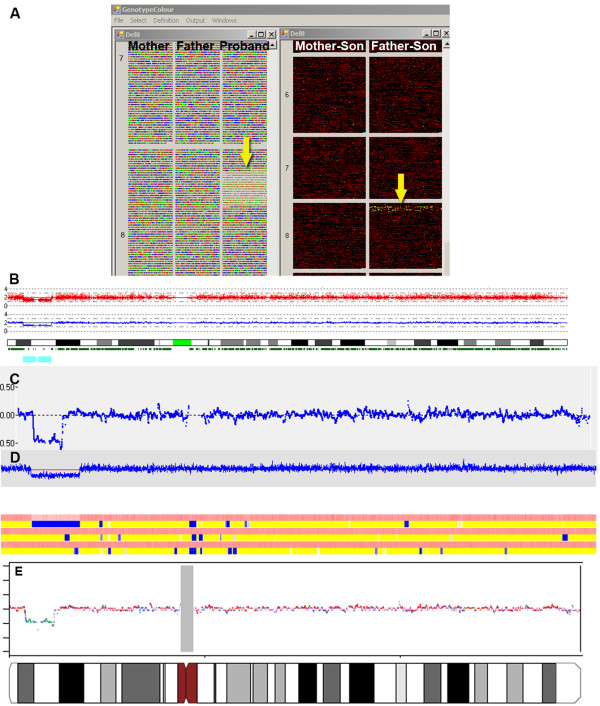
**Trio comparisons**. A) Left-hand side: The portions of chromosomes 7 and 8 of the mother, father and child are displayed. The colour code is the same as that described for Figure 1. The height of the squares is proportional to the CN state. In the proband, a partial deletion of chromosome 8, characterized by the absence of heterozygous genotypes and squares of reduced height, is shown by an arrow. Right-hand side: The genotype comparisons between mother-son and father-son for all SNPs on chromosomes 6, 7 and 8 are displayed. SNPs with identical genotype between the samples are in black, those with one identical allele are in red and those with no identity are in yellow. The presence of yellow squares in the father-son comparison (yellow arrow), combined with the information on the CN state of the trio for the same SNPs (left-hand side, yellow arrow), indicates that the deleted portion of chromosome 8 in the child was of paternal origin. B) C), D) and E) The intensity values and the LOH of the proband chromosome 8 probes are displayed as reported by CNAG, CNAT4, dChip, and SNPscan, respectively.

**Figure 7:**
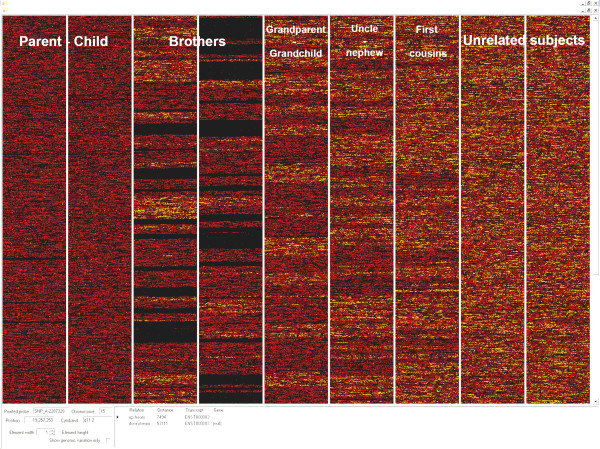
**Two by two comparisons**. Nine "two sample" comparisons between members of the same family with different degrees of kindred and unrelated samples. SNPs with genotypes identical between the samples are in black, those with one identical allele are in red, and those with no identity are in yellow. Only the portion of the genome from 1p36 to 11q14 is displayed. For further explanation, see the text.

**Figure 8:**
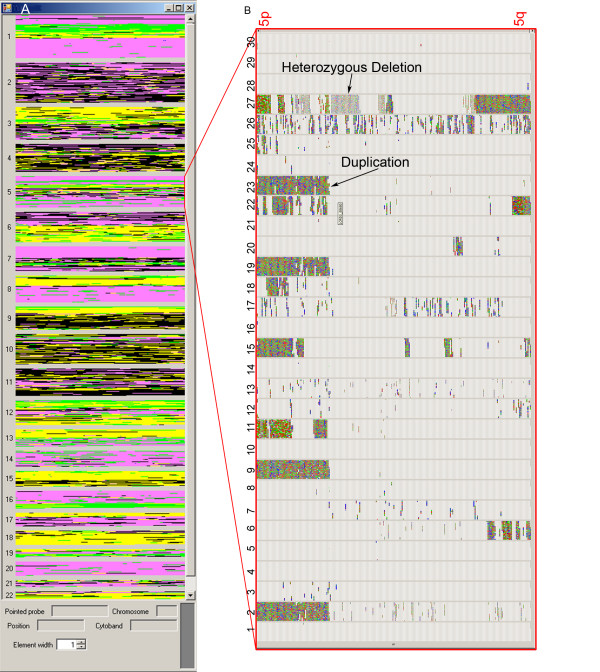
**Comparisons among different tumour samples recovered from the literature**. A) The CN state comparison of 30 tumour specimens is reported. Only the CN state comparison of autosomal chromosomes is shown. The colour code is the same as that described for Figure 6. B) Visualisation of the chromosome 5 SNPs with CN state different from state 2 in 30 tumour samples. The image is rotated by 90° counter clockwise. The left-hand side of the image corresponds to the short arm of chromosome 5, and the right-hand side corresponds to the long arm. For further explanation of the results, see the application section of the text.

Figures 6, 7 and 8 show the visualisation with *GenotypeColour™ *of the Human Mapping chip 250 K data. The three figures are examples of the application of *GenotypeColour™ *to the analysis of a trio, of a large pedigree and of 30 tumour specimens, respectively. In the first example, *GenotypeColour™ *was used to look for possible cryptic alterations in a proband with an apparently normal karyotype (46, XY). Both parents were analysed to verify the origin of possible alterations. For the first analysis, both genotype calls were imported from the Affymetrix GTYPE software and the CN state from the Affymetrix CNATviewer. In the proband, the genotype visualisation showed a region of LOH on the small arm of chromosome 8.

After uploading the CN state, it became clear that the LOH was due to a deletion that was not present in the parents (Figure 6A). The genotype comparisons between the son and his parents highlighted that the chromosome with the deletion was of paternal origin (Figure 6A). The same analysis was performed with CNAT, CNAG, dChip and SNPscan. As shown in Figure 6B–E, all software detected the deletion and showed the deletion and the region of LOH graphically; however, none was able to show the immediate identification of the maternal or paternal origin of the deletion.

In the second example, simultaneous comparisons of different members of the same family were made by assigning three different customised colours to the three possible outcomes (same genotype, one allele equal, no common allele). As shown in Figure 7, the three colours (see the legend) were displayed homogeneously when comparing unrelated subjects, whereas the colours were clustered with different patterns according to the level of kinship in all other cases. For example, in parent-child comparisons, the colour corresponding to no common allele (yellow) was virtually absent, showing that the parents always shared at least one allele with their children for each SNP. The small number of yellow pixels (~0.2%), distributed randomly inside the genome, was compatible with the genotype error rate of the array methodology.

However, if the coloured pixels corresponding to the "AA-BB comparison" are concentrated in a particular chromosomal region, this indicates the presence of potential uniparental disomy (UPD) or CNV in one of the two samples, as in the case of Figure 6A. The comparisons between brothers or sisters were characterized by wide monochromatic regions (black in this example) intercalated among regions with only two colours (black and red) and narrow regions with all three colours. The black clusters correspond to regions where the brothers and sisters share the same portions of paternal and maternal chromosomes; black and red clusters correspond to regions where they share only one parental chromosome; black, red and yellow clusters correspond to regions where they inherited different chromosomes (or chromosome portions) from their parents.

In addition, the boundaries between these regions correspond to recombination breakpoints. In comparisons between grandchild-grandparent, nephew/niece-uncle or cousins, the dichromatic region intercalated between regions with all three colours identify shared inherited haplotypes. The frequency and extension of these dichromatic regions decrease as a function of the degree of kinship up to at least the third generation.

These examples highlight how the *GenotypeColour™ *genotype comparison option can be used to detect kinship and shared haplotypes without calculations or elaborations, using only this new graphical analysis of genotype data. This tool is useful also to identify the origin of the chromosomal alterations in parent-child comparisons and for many other applications, such as linkage and association studies, and population evolution.

In the third example, 30 tumour specimens that belong to the DNA collection of 371 primary lung adenocarcinoma analysed by Weir et al. [9] were compared to detect CNAs. The raw data (.CEL files), freely available, were downloaded from the Broad Institute web site [10] and analysed with Genotyping Console Software to obtain the input files required by *GenotypeColour™*. Comparison of Figures 5 and 8A shows how different is the visualisation outcome for 30 healthy subjects and 30 DNA tumour samples, respectively.

Even if the analysis was done on only 30 out of the 371 samples of the original research [9], most of the large-scale CNAs observed in the original work were clearly identified. It is clear simply from visual inspection that certain chromosomes, such as 2, 4, 10 and 11, are less affected by copy number aberrations in the lung adenocarcinoma DNA cell lines analysed (Figure 8A). In all these chromosomes (as well as in the 3q arm) the predominant colour is black, indicating regions without CNA. The large areas of yellow and pink show that the distribution of deletions and duplications is usually not random and is localized on specific chromosome arms.

The chromosomal distribution of large-scale deletions and amplifications shown by *GenotypeColour™ *agree with those shown by Weir et al., [9] in Figure 1b of their manuscript. Near these preferentially deleted or duplicated regions, others can be seen where both events are present in different samples. These chromosomal regions are reported in green and the largest are those on chromosomes 1 and 16.

The visualisation of the genotype and CN state of all 30 samples allows better definition of breakpoint regions. There is an example in Figure 8B of the genotype and CN state of SNPs on chromosome 5. In addition to the amplification on the small arm (the most frequent aberration in lung adenocarcinoma [9]), rarer smaller aberrations can be visualised. The presence of heterozygous SNP genotypes and CN states equal to state 1 may be an indication of cellular mosaicism for specific chromosome regions (see sample n.27). This example highlights how the CN state comparison option can be used to analyse tumour samples and to identify chromosomal regions preferentially deleted or duplicated in different tumours. This option is also very useful for rapid identification of CN state polymorphisms or CNVs that are present only in certain individuals and might be the cause of specific phenotypes.

## Discussion

*GenotypeColour™ *software represents a new and useful tool for the visualisation of genome variability. Indeed, beside its distinctive features, it condenses in a unique program many of the characteristics of other available software. The main properties of *GenotypeColour*™ are described below.

1. Visualisation of the genotypes of thousands of SNPs in a single computer screenshot. dChip has a similar function; however, the zoom resolution that allows the visualisation of all chromosomes does not allow the visualisation of all SNP genotypes. SNPscan, even if it can discriminate between homozygous and heterozygous genotypes, does not allow visualising and discrimination of all SNPs, since many overlap. Programs such as CNAG and CNAT do not display the genotype of each SNP, even if they do highlight LOH regions.

2. The simultaneous visualisation of genotype and CN state. To our knowledge, the only software allowing simultaneous visualisation of genotype and CN state is SNPscan. In SNPscan, each probe is plotted on a 2D graph with the 23 chromosomes on the *x*-axis and the CN state and the LOH state on the *y*-axis. The homozygous/heterozygous state of the probe is inferred by the colour. *GenotypeColour™*, however, in its maximum zoom-out visualisation, assigns a distinctive pixel to each SNP and depicts them next to one another. This kind of plotting has the advantage of avoiding spatial overlap of probes and improves graphical visualisation. This allows the detection of very small LOH or CNV regions, which can be visualised even better by the "show genomic variation only" option.

3. Sample comparisons: with our software, a "consensus" image is displayed in which a different colour is assigned to each SNP according to the genotype concordance of the samples compared. From these consensus images, regions of identity/diversity among samples are identified immediately. Moreover, the same procedure can be applied to the CN state and to trio comparisons. The only program that can make paired and unpaired comparisons of genotypes between more than two samples is dChip; however, the algorithm used for the comparison is different from ours and at present it does not perform the CN state or trio comparisons.

4. The export of CNV and LOH information in tabular format. *GenotypeColour™ *allows us to export in a table the information of many samples simultaneously (columns) × LOH/CNV regions (rows). This format improves the identification of chromosomal features common to more than one subject, and allows handling of information on CNV and LOH for association studies.

5. The rapid comparison between control and pathologic tissues to identify CNA in different chromosomal regions. This seems to be especially useful in tumours.

6. The visualisation of "no calls" in a different colour can give clear information on the quality of the procedure and on the random distribution and/or clustering of uninformative SNPs.

7. Visualisation of population frequencies. This option could be used as a guide to the ancestry of the analysed samples.

## Conclusion

In conclusion, we have created a program that does not require particular bio-computer notions and is easy to use thanks to its self-explanatory interface and detailed instruction manual. The purpose of *GenotypeColour™ *is to provide a user-friendly tool that can rapidly upload, visualise and compare the huge amounts of data produced by the Affymetrix Human Mapping GeneChips without losing the overall view of the data. Indeed, a general view of results can make biological data easier to understand, followed by analysis with more specialised bioinformatic tools. Only its extensive use by other research groups for specific biological problems will give a more exact view of the potential applications of this new way of visualising DNA genomic data.

## Availability and requirements

**Project name**: *GenotypeColour™*

**Project home page**: 

**Operating system(s)**: Windows XP, Windows 2000, Windows Vista.

**Programming language**: Visual Basic

**Other requirements**: MDAC 2.6 and Microsoft Jet 4.0 Database Engine or Microsoft Access database

**License**: free non-commercial research use license

**Any restrictions to use by non-academics**: Commercial use license can be obtained by contacting the authors.

## Authors' contributions

SB conceived the software, carried out its design and planned and supervised the draft of the manuscript. CM participated in the planning and drafted the manuscript with contribution in the software development. SC developed the algorithm following the instructions of SB and CM. All authors have read and approved the final manuscript.
